# Patient Characteristics of a Telemedicine Clinic for Pediatric and Young Adult Postural Orthostatic Tachycardia Syndrome

**DOI:** 10.3390/jcm15041626

**Published:** 2026-02-20

**Authors:** Jeffrey R. Boris

**Affiliations:** Jeffrey R. Boris, MD LLC, P.O. Box 16, Moylan, PA 19065, USA; jeffreyborismd@earthlink.net; Tel.: +1-610-608-7235

**Keywords:** adolescent, dysautonomia, autonomic nervous system, autonomic dysfunction

## Abstract

**Background**: Postural orthostatic tachycardia syndrome (POTS) includes multiple symptoms and comorbid conditions. Assessment for less recognized symptoms and conditions was performed through a telemedicine-only clinic for adolescent and young adult patients with POTS. **Methods**: A retrospective review of records was performed for information obtained during clinical care. Patients up through the age of 23 years were evaluated, either diagnosing or confirming a diagnosis of POTS, and identifying other symptoms and diagnoses. These data were evaluated for differences, including by sex and presence or absence of joint hypermobility. **Results**: In total, 277 patients met the inclusion criteria. The median age was 16.8 years (IQR 15.2–19.1); 88.1% were female. Suspected mast cell activation syndrome occurred in 70% of patients. Joint hypermobility was found in 78.3% of patients; female patients were more affected (80.3% versus 63.6%); 57.0% had both suspected MCAS and joint hypermobility. Migraine was seen in 51.6% of patients; 57.4% had tension-type headache. Females appeared more likely to have tension headache or both types of headache together, while males seemed more likely to have migraine. Joint hypermobility did not influence headache presence or absence. A history of head trauma/concussion was reported in 39.7% of patients, with 14.4% having vestibular symptoms and 4% having convergence disorder. Without head trauma/concussion, 23.8% of patients reported vestibular symptoms, convergence disorder, or both. **Conclusions**: We report previously unrecognized or poorly described symptoms and conditions accompanying POTS. Recognition of these symptoms and conditions in patients with POTS can allow for more complete evaluation and management of debilitating factors and may give insights into underlying pathophysiologies leading to POTS.

## 1. Introduction

As medical disorders occur, postural orthostatic tachycardia syndrome (POTS) is likely in its relative childhood stage with regard to how its symptoms and associated disorders have been characterized [1,2,3]. Multiple studies have documented signs, symptoms, and comorbid conditions seen with POTS [4,5,6,7], and yet it remains both poorly understood as well as open to further study to better define this syndrome as more concomitant concerns are recognized [8,9,10]. Although POTS is recognized to be a dysautonomia and has been shown, in part, to include decreased cerebral perfusion [11,12] in the setting of increased splanchnic and extremity pooling [13,14], suggesting decreased systemic venous return and cardiac output as part of its pathophysiology, the relationship and influences of other comorbidities, such as connective tissue disorders and mast cell activation syndrome [4,5,6,7], are not well understood.

In the management of POTS, clinical care varies from single-provider care to multidisciplinary clinics. In October 2020, the present author opened a telemedicine-only consultation clinic for the care of children and young adults with POTS. After having cared for over 270 patients for over five years since the clinic opened, some unique patient characteristics not previously well documented in the medical literature began to emerge, which are shared here. These other clinical findings, which have not been routinely described in the setting of POTS, were noted as the historical evaluation was widened. A review of these less frequently reported findings was undertaken in order to further recognize and acknowledge these less appreciated aspects which may feed into the underlying pathophysiology of POTS.

## 2. Materials and Methods

Pediatric, adolescent, and young adult patients up to the age of 23 years were evaluated and managed in a telemedicine-only consultation clinic operated by the author. Patients self-referred for evaluation through the clinic website. The clinic is conducted virtually across multiple states in the United States, with multiple state licensures, in order to be able to conduct telemedicine remotely. Telemedicine visits were conducted with a secure, HIPAA-compliant platform. The inclusion criteria for this study included at least 3 months of symptoms of chronic orthostatic intolerance plus a sustained increase in upright heart rate of at least 30 beats per minute in the first 10 min of standing after at least 5 min supine [1]. An observed standing test with minimal stimulation was performed, with heart rate measured by home blood pressure cuff, finger pulse oximetry, or electronic heart rate monitor. If patients had already had documentation of a prior tilt table test or 10 min standing test that was consistent with POTS, those results were utilized for diagnosis, and testing was not repeated. Patients with other etiologies which could cause these findings, who did not meet these criteria, or who had other autonomic disorders, were excluded. Patients were treated with a combination of nonpharmacologic interventions, medications, and exercise to attempt to control any symptoms that were deemed to be interfering with the patients’ activities of daily living.

Diagnosis of suspected mast cell activation syndrome (MCAS) was made based on the presence of a combination of multiple symptoms from at least two systems, including cutaneous (pruritus, urticaria, flushing, dermatographia, and oral allergy symptoms), gastrointestinal (nausea, bloating, and diarrhea), and urologic (repeated dysuria and urinary frequency in the absence of urinary tract infection suggestive of interstitial cystitis); cardiovascular manifestations, including tachycardia, palpitations, and orthostatic intolerance, were not included as one of the involved systems, as these symptoms are nonspecific and can be seen in POTS in the absence of MCAS or in MCAS in the absence of POTS. Laboratory analysis for mast cell-associated inflammatory mediators was not performed. Diagnosis of joint hypermobility syndromes, including hypermobile Ehlers–Danlos syndrome (hEDS), generalized hypermobility spectrum disorder (G-HSD), and localized hypermobility spectrum disorder (L-HSD), was based on the 2017 diagnostic criteria [15,16]. A Beighton score was performed on all patients [16] by visual assessment and estimation of the specific angles involved in the diagnosis of hypermobility. Patients were not assisted by parents for this evaluation. Diagnoses of autism spectrum disorder and attention deficit hyperactivity disorder, as previously conferred by the patients’ primary care or specialty care clinicians, were queried. Diagnosis of headache types (migraine or tension-type), autism spectrum disorder, history of head trauma or concussion, vestibular symptoms, and convergence insufficiency was obtained by clinical history. Statistical analysis included percentage calculations, which were performed with Microsoft Excel 2010 (Microsoft, Redmond, WA, USA), and chi-square analysis, as well as the Kolmogorov–Smirnov test for normality, performed using the website, Social Science Statistics (www.socscistatistics.com). Institutional Review Board exemption determination was granted by Castle IRB (Castle IRB, Chesterfield, MO, USA) for this study after the background of the study methods was reviewed as study POTS-001.

## 3. Results

Out of 317 patients evaluated in the clinic from October 2020 to June 2025, 277 met the inclusion criteria. The analysis demonstrated that the patient ages for the total sample and for females were not normally distributed, with the test statistic for all patients at 0.10 (*p* = 0.0066) and for female patients at 0.097 (*p* = 0.019); male patient ages were normally distributed, with a test statistic of 0.12 (*p* = 0.65). The median age of the total sample was 16.8 years (interquartile range 15.2–19.1), and 244 (88.1%) of patients were females (Table 1). Comorbid suspected MCAS was seen in 70.0% of patients. Joint hypermobility was diagnosed in 78.3% of patients and seen in 80.3% of female patients and 63.6% of male patients. The majority of hypermobility cases were diagnosed as G-HSD (63.6% of all hypermobile patients). L-HSD and hEDS comprised 19.8% and 16.6% of all hypermobile patients, respectively. A total of 158, or 57.0%, of all patients with POTS had both suspected MCAS and joint hypermobility. Comorbid autism spectrum disorder was seen in up to 9.0% of patients, and attention deficit hyperactivity disorder was noted in 39.4% of patients.

Over half of the patients (51.8%) had migraine, and over half (57.4%) had tension-type headache (Table 2). Females appeared more likely to have tension headache (60.2% vs. 36.4%) as well as co-occurring migraine and tension headache (36.9% vs. 15.2%), while males seemed more likely to have migraine (30.3% vs. 15.6%). Around one-third of the patients had both migraine and tension-type headaches. Although not necessarily connected as vestibular migraine, one-third of the patients had a history of migraine plus vestibular symptoms. When joint hypermobility was considered, 51.6% of hypermobile patients versus 51.7% of non-hypermobile patients had migraine, and 58.1% of hypermobile patients versus 55.0% of non-hypermobile patients had tension-type headache (Table 3). As a percentage of all patients with POTS, female hypermobile patients looked more likely to have migraine than males (42.6% vs. 24.2%), as well as to have tension-type headache (48.4% vs. 24.2%).

As many as 39.7% of patients reported a prior history of head trauma or concussion, with 14.4% of these patients having concurrent vestibular symptoms and 4% having convergence disorder at the time of evaluation; thus, approximately 18% of patients had either of these symptoms, and about 12% of patients had both of these symptoms after a history of head trauma (Table 3). Over half of the patients (55.2%) reported vestibular symptoms, and 36.1% reported convergence disorder, regardless of whether there had been a history of head trauma or concussion or not. However, 23.8% of patients had vestibular symptoms and/or convergence disorder without a known history of head trauma or concussion, and nearly 8% of patients had both of these symptoms together.

## 4. Discussion

Patients with POTS can have both multiple symptoms and multiple comorbid conditions [4,5,17], which have been recognized for several years now [1,5]. However, these newer findings of other symptoms and comorbidities shed further light onto an increasing understanding of the wide range of various conditions that accompany POTS, and confirm the old saying, “if you don’t look, you won’t find it.”

We had previously published that 21.5% of these patients also had self-reported mast cell activation disorder [18]; however, with as many as 70% of patients having findings consistent with MCAS, further attention will need to be paid to assessing symptoms of this condition, as well. Since laboratory testing for inflammatory mediators was not performed, a diagnosis of MCAS could not be given to the patients seen in this clinic. However, patients were treated with various anti-inflammatory therapies, such as antihistamines, oral cromolyn, and omalizumab, as appropriate, with all patients reporting clinical improvement with the addition of some or all of these treatments. A prior study had shown that the prevalence of MCAS in the setting of postural orthostatic tachycardia syndrome was 42%, which is lower than our findings [19], although a more recent study suggested that MCAS prevalence in 100 patients with POTS was as high as 87% when only clinical criteria were utilized for diagnosis [20], which is more in line with our findings. It is interesting to note that in this more recent study, only 2% of patients with MCAS would have been recognized utilizing consensus-1 criteria, and 37% would be recognized using consensus-2 criteria, suggesting that there needs to be a better understanding of MCAS and its diagnostic criteria, as this is increasingly seen in the setting of POTS. As there is an overlap of symptomatology in POTS and mast cell activation, including gastrointestinal findings such as nausea, abdominal pain, and bloating, as well as cardiovascular findings in which inflammatory mediators released by mast cells can increase vascular permeability, reduce intravascular volume, and cause cerebral hypoperfusion with symptoms of orthostasis, attention may also need to be paid to attempting to parse the specific etiologies of these symptoms, with appropriate pharmacologic targeting to not only manage POTS symptoms but those of MCAS as well.

Joint hypermobility in association with POTS has been recognized for a while [4,21]. We had also previously reported that as many as 61% of these patients had joint hypermobility [22]. The finding that 78% of this present cohort has hypermobility, then, is likely similar. The appearance of a greater presence of hypermobility in female patients is not surprising, as this has also been demonstrated previously [23]. However, it is notable that nearly two-thirds of male patients also had joint hypermobility; for comparison, the prevalence of joint hypermobility in the general population ranges from 2 to 57%, although the prevalence that most studies referenced ranged between 6 and 39% [24]. Of note, due to the substantial overrepresentation of females versus males, statistical significance between sexes could not be evaluated.

Autism spectrum disorder has previously been associated with POTS [25], with as many as 9 out of 28, or 32%, of autistic patients also having POTS. Attention deficit hyperactivity disorder has also been seen with POTS [26]. As well, autism spectrum disorder and attention deficit hyperactivity disorder have been associated with joint hypermobility [27]. In this series, 9% of patients with POTS also had a diagnosis of autism spectrum disorder, and nearly 40% of patients had attention deficit hyperactivity disorder. Comparatively, the published prevalence of autism in the United States from 2022 is 3.2% [28], and the prevalence of attention deficit hyperactivity disorder from 2017 to 2022 is 10.08–10.47% [29]. It is notable that in a recent study assessing possible genetic markers in POTS, a significant subset of mutations noted were associated with autism spectrum disorder [30]. Further confirmatory and proteomic studies demonstrating the association of POTS and neurodivergence would be beneficial, as they bring up the question of whether there is a true genetic linkage as well as a possible pathophysiologic mechanism between these two entities.

Migraine has previously been reported in the setting of POTS [31,32]. Our study demonstrates that approximately half of the patients have migraine. However, we also demonstrate that 57.4% of patients have tension-type headache, which has not been previously reported. We also noted that over one-third of the patients had complaints of both migraine and tension-type headache separately. Anecdotally, we noted that the tension-type headaches would frequently occur daily, while the migraines would be less frequent. Female patients seemed more likely to have tension-type headache, while males appeared to be more likely to have migraine. Females also appeared to report having both migraine and tension headaches more frequently. Although we did demonstrate that nearly one-third of the patients with migraine also had vestibular symptoms, which could imply the presence of vestibular migraine, we anecdotally found that the large majority of patients found that their episodes of migraine and vertigo were separate and unrelated. For comparison, the reported prevalence of migraine is 15.3% (9.7% of males, 20.7% of females) [33], and that of tension-type headache is 38.3% [34], although the prevalence of chronic tension-type headache (at least 15 days per month or continuous), which all patients in this study reported, is 2–4% [35]. Anecdotally, patients were referred for osteopathic manipulation therapy (OMT) for the management of tension-type headache, which was felt to be effective in relieving these symptoms in many patients. OMT has previously been reported for use in the management of POTS symptoms [36], although not specifically for tension-type headache in the setting of POTS. The frequency and outcomes of patients who received OMT were not specifically systematically tracked in our study, however.

When assessing for the effect of joint hypermobility on the presence of either migraine or tension-type headache, the only differences found were that female patients with POTS with joint hypermobility also appeared more likely than male patients to have either migraine or tension-type headache (Table 3). Interestingly, although 40.4% of patients with hypermobility had migraines, while 11.2% of non-hypermobile patients had migraines, 37.9% of hypermobile patients did not have migraines, and 10.5% of non-hypermobile patients also did not have migraines. Similar findings were seen in the setting of tension-type headache. Thus, the presence or absence of joint hypermobility did not influence the presence or absence of headaches in this patient population.

Concussion is a known trigger for POTS [4,37]. In this series of patients, 39.7% of patients reported a prior history of head trauma or concussion, although it was not necessarily the trigger for the onset of their POTS in most. Both vestibular dysfunction and convergence disorder are recognized components of post-concussion syndrome [38]; in this series, 14.4% and 4.0% of patients, respectively, reported these persistent conditions with a prior history of head trauma at the time of initial evaluation, with 11.9% of patients reporting having both of these concerns. However, even in the absence of head trauma or concussion, over 20% of patients with POTS reported vestibular symptoms, and about 3% reported convergence disorder; nearly 8% of patients had both of these problems, with no sex differences noted. Vestibular dysfunction with POTS has been previously described, with a team from Korea noting increased sympathetic tone in the setting of exaggerated ocular vestibular-evoked myogenic potentials [39,40]. As previously mentioned, it can also be seen in the setting of vestibular migraine [41]. Interestingly, the reported prevalence of vestibular dysfunction is 30.4% [42], and the prevalence of convergence disorders is 4.2–17.6% [43], both of which are similar to those of our patient population. These findings bring up a couple of important points. First, both patients and clinicians often utilize the term “dizziness” when describing symptoms of disorientation. However, dizziness is a nonspecific term that does not specifically connote the probable underlying complaint [44]. When more specific terms are used, such as “lightheadedness” and “vertigo,” and these terms are appropriately described for patients, they are more able to identify their specific symptoms and to be treated more suitably, as lightheadedness is more consistent with orthostatic intolerance while vertigo is seen in the setting of vestibular dysfunction or persistent perceptual postural dizziness. This then allows for better screening and directed care, including vestibular therapy for those affected with vertigo, and vision therapy and/or the use of glasses with prisms in the management of convergence disorder. The other issue is that the presence of a significant amount of vestibular symptoms in patients with POTS without head trauma may give some insight into the pathophysiologic process underlying POTS. Also, of note, the previously mentioned study of putative genetic markers for POTS also listed findings associated with a mutation in the OTOG gene, which is associated with vestibular dysfunction [30].

There are some limitations in this study. These include using a 30 beats per minute heart rate threshold for the diagnosis of postural orthostatic tachycardia syndrome in patients under age 20. However, numerous patients had been previously diagnosed after demonstrating a positive tilt table test or ten-minute standing test in an outside facility. These facilities used a 40 beats per minute cutoff. We had previously published data demonstrating that there was no difference in symptom type or severity in adolescents with a 30-beat-per-minute threshold versus a 40-beat-per-minute threshold on a ten-minute standing test [45]. Furthermore, at least three studies have demonstrated that tilt table testing induces a higher heart rate response as compared to the standing test [46,47,48]. Prior to the 2011 consensus statement that established 40 beats per minute as the adolescent threshold (with no documentation as to why this number was chosen) [49], 30 beats per minute was used for diagnosis for all patients. In fact, the authors stated that it was NOT an evidence-based clinical guideline. In my prior practice at a large children’s hospital, 30 beats per minute was also used as the diagnostic cutoff, long before the subsequent change.

As well, there is a disagreement in the medical literature regarding the use of the consensus-2 diagnostic criteria for mast cell activation syndrome [50], although in this patient population, a formal diagnosis of mast cell activation disorder could not specifically be given, as testing was not performed, so these patients could only be labeled as having suspected MCAS. As mentioned, with the overrepresentation of female patients combined with a relatively small male cohort, statistical evaluation between sexes could not be performed. Although we demonstrated evidence of multiple new symptoms and conditions in these patients, neither temporality nor causality can be demonstrated simply by association. Lastly, the patients seen through this telemedicine clinic may be a skewed sample, as they are patients who have previously typically not had successful therapy for their symptoms of chronic orthostatic intolerance, as well as those who can afford to be seen in this clinic without insurance coverage. However, multiple patients in this practice were able to get partial or complete reimbursement through their insurance provider after submitting an invoice demonstrating ICD-10 as well as evaluation and management codes.

These data show that inflammatory symptoms that are likely consistent with MCAS occur much more frequently with postural orthostatic tachycardia in adolescents and young adults than was previously recognized, and that joint hypermobility remains very common in this population, as well. Further research among other cohorts, specifically to confirm the prevalence of mast cell activation syndrome, is warranted. However, increased attention to therapies for mast cell activation may further help to reduce symptoms associated with postural orthostatic tachycardia syndrome. As well, ensuring appropriate physical therapy support for patients with hypermobility to teach them to protect and to stabilize their joints remains important to prevent joint pain and arthritis [51].

Tension-type headache occurs quite frequently in these patients and can occur separately, but concurrently, with migraine. Neurodivergent diagnoses, such as autism spectrum disorder and attention deficit hyperactivity disorder, are also frequently seen. And, knowledge of the various comorbidities seen in the setting of POTS, with appropriate ascertainment of history and symptomatology, may lead to more successful control of patients’ symptoms and more ability of patients to have improved quality of life and functionality. Finally, subsequent research demonstrating temporal and, potentially, causal aspects of these findings may be able to give further insight into the pathophysiologies underlying this chronic and debilitating disorder.

## Figures and Tables

**Table 1:** Comorbid conditions of patients with POTS *n* (%).

	Total	Female	Male
Patients	277	244 (88.1)	33 (11.9)
Comorbid Conditions			
Suspected MCAS	194 (70.0)	174 (71.3)	20 (60.6)
Any JH	217 (78.3)	196 (80.3)	21 (63.6)
hEDS	36 (16.6)	32 (16.3)	4 (19.0)
G-HSD	138 (63.6)	126 (64.3)	12 (57.1)
L-HSD	43 (19.8)	38 (19.4)	5 (23.8)
SMCAS plus ANY JH	158 (57.0)	143 (58.6)	15 (45.5)
SMCAS plus hEDS	34 (12.3)	31 (12.7)	3 (9.1)
SMCAS plus G-HSD	96 (34.7)	87 (35.7)	9 (27.3)
SMCAS plus L-HSD	28 (10.1)	25 (10.2)	3 (9.1)
			
ASD	25 (9.0)	23 (9.4)	2 (6.1)
ADHD	109 (39.4)	97 (39.8)	12 (36.4)

POTS: Postural Orthostatic Tachycardia Syndrome; MCAS: Mast Cell Activation Syndrome; JH: Joint Hypermobility; hEDS: Hypermobile Ehlers–Danlos Syndrome; G-HSD: Generalized Hypermobility Spectrum Disorder; L-HSD: Localized Hypermobility Spectrum Disorder; SMCAS: Suspected MCAS; ASD: Autism Spectrum Disorder; ADHD: Attention Deficit Hyperactivity Disorder. All percentages shown are as a percentage of patients with POTS (total, female, or male).

**Table 2:** Comorbid symptoms of patients with POTS *n* (%).

	Total	Female	Male
History of migraine	143 (51.6)	128 (52.5)	15 (45.5)
History of tension-type headache	159 (57.4)	147 (60.2)	12 (36.4)
History of migraine alone	48 (17.3)	38 (15.6)	10 (30.3)
History of tension-type headache alone	64 (23.1)	57 (23.4)	7 (21.2)
History of both migraine and tension-type headache	95 (34.3)	90 (36.9)	5 (15.2)
History of migraine and vestibular symptoms	90 (32.5)	82 (33.6)	8 (24.2)
			
History of head trauma/concussion	110 (39.7)	102 (41.8)	8 (24.2)
History of vestibular symptoms	153 (55.2)	138 (56.6)	15 (45.5)
History of convergence disorder	74 (36.1)	69 (28.3)	5 (15.2)
History of head trauma plus vestibular symptoms	40 (14.4)	37 (15.2)	3 (9.1)
History of head trauma plus convergence disorder	11 (4.0)	10 (4.1)	1 (3.0)
History of head trauma plus either vestibular symptoms or convergence disorder	51 (18.4)	47 (19.3)	4 (12.1)
History of head trauma plus both vestibular symptoms and convergence disorder	33 (11.9)	30 (12.3)	3 (9.1)
History of head trauma without vestibular symptoms or convergence disorder	26 (9.4)	25 (10.2)	1 (3.0)
History of vestibular symptoms without head trauma or convergence disorder	58 (20.9)	49 (20.1)	9 (27.3)
History of convergence disorder without head trauma or convergence disorder	8 (2.9)	7 (2.9)	1 (3.0)
History of vestibular symptoms and/or convergence disorder without head trauma	66 (23.8)	56 (23.0)	10 (30.3)
History of both vestibular symptoms and convergence disorder without head trauma	22 (7.9)	22 (9.0)	0

**Table 3:** Influence of Joint Hypermobility on Comorbid Headache *n* (%).

	Total JH 217 No JH 60 POTS 277	Female JH 196 No JH 48 POTS 244	Male JH 21 No JH 12 POTS 33	*p* Value with JH vs. Without JH
History of migraine plus JH (percentage of patients with POTS with JH) (percentage of all patients with POTS)	112 (51.6) (40.4)	104 (53.1) (42.6)	8 (38.1) (24.2)	0.99
History of migraine without JH (percentage of patients with POTS without JH) (percentage of all patients with POTS)	31 (51.7) (11.2)	24 (50.0) (9.8)	7 (58.3) (21.2)	
History of tension-type headache plus JH (percentage of patients with POTS with JH) (percentage of all patients with POTS)	126 (58.1) (45.5)	118 (60.2) (48.4)	8 (38.1) (24.2)	0.67
History of tension-type headache without JH (percentage of patients with POTS without JH) (percentage of all patients with POTS)	33 (55.0) (11.9)	29 (60.4) (11.9)	4 (33.3) (12.1)	

JH: joint hypermobility; POTS: postural orthostatic tachycardia syndrome.

## Data Availability

The raw data supporting the conclusions of this article will be made available by the authors on request.
